# Bearing Anomaly Detection Method Based on Multimodal Fusion and Self-Adversarial Learning

**DOI:** 10.3390/s26020629

**Published:** 2026-01-17

**Authors:** Han Liu, Yong Qin, Dilong Tu

**Affiliations:** The State Key Laboratory of Rail Traffic Control and Safety, Beijing Jiaotong University, Beijing 100044, China; liuhan@cqsf.com (H.L.); 23115092@bjtu.edu.cn (D.T.)

**Keywords:** bearing abnormal detection, multimodal fusion, time series data augmentation, self-adversarial training

## Abstract

In the context of bearing anomaly detection, challenges such as imbalanced sample distribution and complex operational conditions present significant difficulties for data-driven deep learning models. These issues often result in overfitting and high false positive rates in complex real-world scenarios. This paper proposes a strategy that leverages multimodal fusion and Self-Adversarial Training (SAT) to construct and train a deep learning model. First, the one-dimensional bearing vibration time-series data are converted into Gramian Angular Difference Field (GADF) images, and multimodal feature fusion is performed with the original time-series data to capture richer spatiotemporal correlation features. Second, a composite data augmentation strategy combining time-domain and image-domain transformations is employed to effectively expand the anomaly samples, mitigating data scarcity and class imbalance. Finally, the SAT mechanism is introduced, where adversarial samples are generated within the fused feature space to compel the model to learn more generalized and robust feature representations, thereby significantly enhancing its performance in realistic and noisy environments. Experimental results demonstrate that the proposed method outperforms traditional baseline models across key metrics such as accuracy, precision, recall, and F1-score in abnormal bearing anomaly detection. It exhibits exceptional robustness against rail-specific interferences, offering a specialized solution strictly tailored for the unique, high-noise operational environments of intelligent railway maintenance.

## 1. Introduction

As a key component that supports the weight of the train and ensures its smooth operation, the health condition of train bearings is directly related to the safety and efficiency of railway transportation. Once a bearing abnormality occurs, it may lead to catastrophic derailment accidents, resulting in significant loss of life and property [1,2,3].

Bearing vibration data serves as a critical indicator of bearing health status. Such data is typically collected in real-time via sensors during train operation. During acquisition, vibration signals are influenced by multiple complex and non-stationary external factors, such as ambient temperature, wind force, train speed, and travel distance. Early data-driven approaches primarily relied on signal processing indicators, such as blind diagnostic metrics which utilize statistical properties like kurtosis or the smoothness index to detect impulses without prior knowledge [4,5]. However, these traditional threshold-based anomaly detection methods are often unreliable, prone to false alarms or missed detections, highlighting the importance of developing more intelligent anomaly detection methods that can adapt to complex operational conditions.

While deep learning has demonstrated significant potential in abnormal bearing detection, this field still faces several core challenges: First, data scarcity and imbalance. Although vast amounts of normal operational data are available, actual anomaly samples are extremely rare due to strict maintenance protocols in the railway industry. This “long-tail distribution” characteristic makes it difficult for data-driven models to learn effective generalized features from limited abnormal samples, often leading to overfitting on normal operational data. Second, signal complexity and nonlinearity. Bearing vibration time-series signals are influenced by multiple environmental and operational factors, exhibiting significant nonlinear and non-stationary behavior. Finally, model robustness issues. Models trained on clean laboratory data often experience a sharp decline in performance when exposed to unpredictable, noisy, and disturbed real-world data. Therefore, enhancing the generalization capability and robustness of models to ensure reliable performance after deployment is a critical research direction.

Abnormal bearing vibration detection methods can be broadly categorized into two types: physics-based models and data-driven models. Physics-based models, such as thermodynamic behavior analysis and finite-element analysis (FEA), predict bearing behavior by simulating its physical characteristics. However, these methods are computationally expensive and heavily rely on precise physical parameters, making it difficult for them to adapt to dynamic changes during train operation. In contrast, data-driven methods leverage historical data to learn anomaly patterns, eliminating the need for complex physical modeling, as comprehensively reviewed in recent roadmaps for machine fault diagnosis [6]. Early data-driven approaches primarily relied on traditional machine learning algorithms, such as Support Vector Machine (SVM). In recent years, with the advancement of big data and computational power, deep learning models, such as Long Short-Term Memory (LSTM) and Gated Recurrent Unit (GRU) networks [7,8,9], have gained significant attention due to their exceptional ability to process time-series data and capture long-term dependencies. Specifically, in the context of bearing diagnostics and image analysis, converting 1D signals into 2D images (e.g., GADF [10]) has become a hotspot. For instance, Spirto et al. [11] compared SDP-CNN with Time–Frequency approaches, and Jiang et al. [12] utilized Convolutional Capsule Networks for bearing diagnosis.

However, despite these advancements, critical research gaps remain when applying these methods to actual railway maintenance. First, severe class imbalance poses a major hurdle; standard deep learning models tend to become biased toward the majority “normal” class, leading to high false-negative rates for rare but catastrophic faults. Second, feature extraction capability is often limited by a unimodal approach. Relying solely on 1D time-series data may miss complex nonlinear correlations, while using only 2D image representations can lose high-frequency temporal trends. Finally, robustness in high-noise environments is frequently overlooked. Existing models often lack intrinsic mechanisms to resist non-stationary interferences (such as wheel–rail impacts), resulting in rapid performance degradation when deployed in real-world scenarios. Consequently, there is an urgent need for a unified framework that can synergistically fuse multimodal information and enforce robust feature learning to overcome these specific limitations.

To address data scarcity and complexity, recent studies utilize spatiotemporal multi-sensor fusion [13,14] and progressive contrastive learning [15,16] to enhance robustness. Inspired by these advancements, this paper proposes a novel deep learning training framework that integrates multimodal data fusion, data augmentation techniques, and the SAT mechanism to mitigate issues arising from data scarcity, signal complexity, and insufficient model robustness. The main contributions of this study can be summarized as follows:

1. This study is the first to systematically integrate one-dimensional bearing vibration time-series data with their corresponding 2D GADF image representations, overcoming the limitations of single-modal approaches. This method combines the uncompressed linear temporal features from the original time-series data with the nonlinear spatiotemporal correlation features encoded in GADF images, constructing a more comprehensive and discriminative feature set.
2. A series of augmentation techniques specifically designed for time-series data is applied to expand the training dataset. This effectively alleviates the small-sample problem, significantly improves model performance, and enhances the model’s robustness to real-world variations.
3. A powerful training strategy, namely self-adversarial training, is adopted to further improve the model’s generalization capability and resistance to minor perturbations. This approach enhances the model’s stability and reliability in dealing with uncertain and noisy data scenarios.

The remainder of this article is organized as follows: Section 2 elaborates on the proposed deep learning framework, detailing the theoretical principles of the Gramian Angular Difference Field (GADF) transformation, the architecture for multimodal feature extraction and fusion, the specific time-series data augmentation strategies, and the implementation of the SAT mechanism. Section 3 presents the experimental case study, which includes a detailed description of the real-world railway dataset and experimental setup, followed by a comprehensive analysis of the results, visual interpretations, ablation studies, and noise robustness validation. Finally, Section 4 summarizes the research conclusions, discusses the limitations of the current methodology, and outlines potential directions for future work.

## 2. Proposed Method

**Figure 1:**
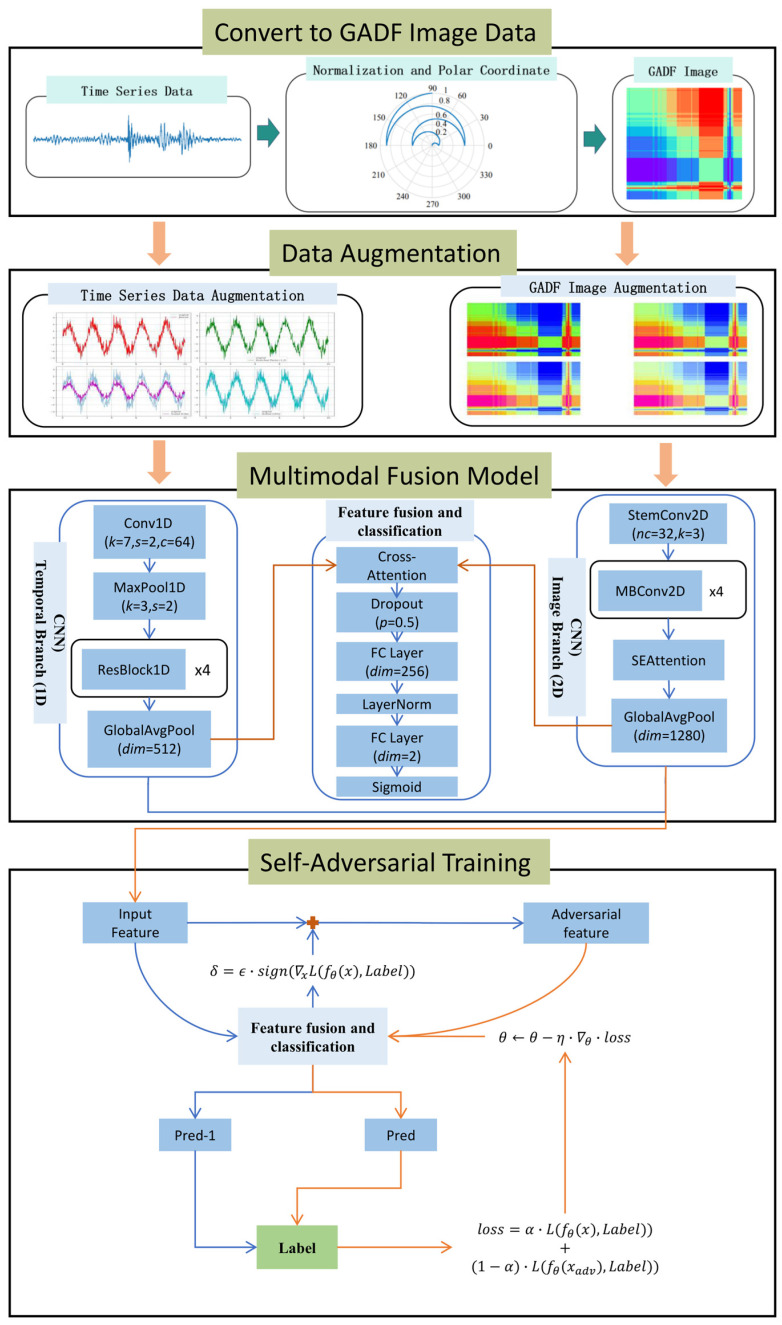
Architecture of the framework.

This section elaborates on the proposed deep learning framework for bearing anomaly detection. Its core innovation lies in the organic integration of multimodal feature representation for time-series data, data augmentation, and SAT. The workflow begins with raw one-dimensional bearing vibration time-series data, which is processed through two parallel branches for feature extraction. The extracted features are then fused, and the entire model is optimized through a SAT loop. The architecture of the framework is shown in Figure 1.

### 2.1. GADF

A major challenge in time series analysis lies in its nonlinearity and non-stationarity. To leverage powerful CNN models developed for image processing in the field of computer vision, researchers have begun exploring the conversion of one-dimensional time series signals into 2D images, enabling analysis with sophisticated image classifiers like Convolutional Neural Networks (CNNs). This has emerged as a promising and effective approach.

Beyond common time-frequency analysis methods, several specialized imaging techniques for time series data have been developed, such as Gramian Angular Fields (GAF), and its improved variants for fault identification [17], Recurrence Plots [18] and Markov Transition Fields [19]. Among these, GADF method stands out. It maps one-dimensional time series data into a polar coordinate system and generates an image by calculating angular relationships between data points.

The process involves first normalizing the time series data and mapping each point to polar coordinates, where each value corresponds to a unique angle and radius. Then, a matrix is constructed by computing the sine values of the angular differences between all pairs of data points. This matrix is ultimately transformed into a 2D image. Essentially, this image acts as a “fingerprint” that encodes nonlinear relationships between data points and periodic information of the time series, revealing complex patterns hidden in the original signal.

GADF imaging is a powerful feature engineering tool. For instance, an upward trend in the original signal might be compressed into a distinct texture in the GADF image. While CNNs can learn and recognize such textures, this approach involves a critical trade-off: it may lose important linear temporal information from the original time series, such as seasonality, trends, or specific temporal patterns. For example, a GADF image can represent pairwise relationships between data points but cannot directly reflect the absolute chronological order of the original series.

**Figure 2:**
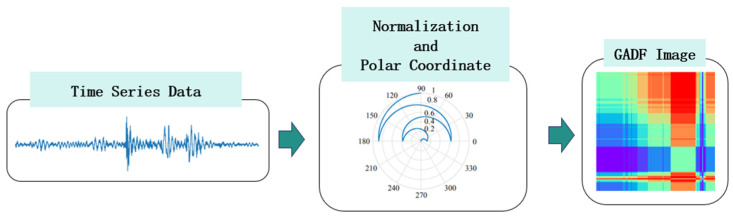
The key workflow of GADF transformation.

Therefore, the core idea of this study is to complement the nonlinear relational patterns captured by GADF images with the linear temporal features of the original time series data, thereby achieving a more comprehensive and robust data representation. The key workflow of GADF transformation is shown in Figure 2.

As shown in the figure, converting time series data into a GADF image primarily involves four steps: normalization, polar coordinate transformation, GADF matrix construction.

1. Normalization: The raw one-dimensional time series is normalized to the range [−1, 1]. This step eliminates the dimensional influence of the original data and ensures that the subsequent calculated angle values fall within the domain of the arccosine function. The normalization formula is as follows:

(1) $${\tilde{x}}_{i}=\frac{2\cdot (x_{i}-min(X))}{max(X)-min(X)}-1$$

2. Polar Coordinate Transformation: Each normalized data point is mapped to polar coordinate. The polar coordinate transformation formula is as follows:

(2) $$\phi_{i}=arccos({\tilde{x}}_{i})$$

3. GADF Matrix Construction: Based on the polar coordinate representation, the sine of the angular difference between every two time points *i* and *j* is calculated to fill the (*i*, *j*) element of the GADF matrix. The mathematical definition of the GADF matrix is as follows:

(3) $${GADF}_{ij}=sin(\phi_{i}-\phi_{i})$$

4. Image Generation: The resulting matrix is visualized as a 2D image (typically in grayscale, heatmap, or colormap), where the pixel intensities represent the magnitude of the angular differences, forming a structured representation of the original time series.

### 2.2. Multimodal Feature Extraction and Fusion

Multimodal data fusion refers to the integration of information from multiple data types (e.g., text, images, audio, and sensor data) to construct richer and more comprehensive AI models. While many studies typically fuse data from different sensors (e.g., vibration, acoustics, and temperature), our approach innovatively creates two modalities from a single sensor data source. Based on the stage at which fusion occurs, multimodal fusion strategies can be categorized into data-level (early fusion), feature-level (intermediate fusion), and decision-level (late fusion).

1. Early Fusion

This approach integrates raw or low-level features into a single input vector prior to feature extraction. It requires modalities to be highly correlated and strictly synchronized. If features are not properly normalized, modality-specific nuances may be lost, potentially undermining the model’s ability to capture fine-grained distinctions.

2. Intermediate Fusion

This is the most widely adopted strategy. It first processes each modality independently into latent feature representations, which are subsequently fused. This approach effectively captures rich interactions between modalities, thereby fully leveraging their complementary information.

3. Late Fusion

This method processes each modality independently and combines their respective predictions at the decision level. It is straightforward to implement and exhibits robustness to missing data. However, it inherently fails to capture complex inter-modal interactions during processing.

This study adopts feature-level fusion [20], as it best aligns with the nature of our data. The GADF image and the raw time-series data are not entirely independent modalities but rather two complementary views of the same physical process. The GADF image encodes nonlinear relationships between data points, while the raw time-series data preserves their linear temporal order.

Simply concatenating these two modalities at the input stage (early fusion) may fail to fully capture their underlying interdependencies. In contrast, by fusing them after feature extraction and incorporating a subnetwork based on an attention mechanism, the model can dynamically weigh the importance of features from each modality. This enables the learning of a synergistic and more robust unified representation.

**Figure 3:**
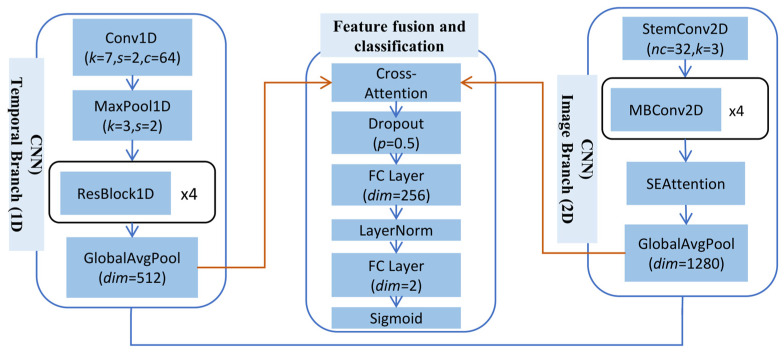
The network structure for multimodal fusion.

The network structure for multimodal fusion is shown in Figure 3. A dual-branch deep neural network is employed to independently extract features from the raw time-series data and the GADF images.

Temporal Branch (1D CNN): This branch is responsible for extracting features from the raw time-series data. Its architecture consists of multiple 1D convolutional layers, activation functions (e.g., ReLU), and pooling layers. The 1D convolutional kernels slide along the temporal dimension to capture local temporal patterns and critical variations in the signal.

Image Branch (2D CNN): This branch is designed to extract features from GADF images, similarly to approaches utilizing vibration images for bearing diagnosis [21]. Its architecture comprises multiple 2D convolutional layers, activation functions, and pooling layers. The 2D convolutional kernels learn spatial patterns in the images, which correspond to complex global temporal correlations and periodic information in the original time series.

After independent feature extraction by the two branches, the feature vectors from the temporal branch and the image branch are concatenated. To move beyond simple linear aggregation, this study further introduces an attention mechanism-based subnetwork [22]. This subnetwork takes the concatenated feature vector as input and learns to assign dynamic weights to each feature. This enables the model to automatically identify and prioritize features most critical to the diagnostic task. For example, in certain fault modes, nonlinear correlations in GADF images may be more important than raw temporal trends. This adaptive fusion strategy ensures the model effectively leverages complementary information from both modalities, avoiding performance bottlenecks associated with straightforward concatenation. The fusion method allows the model to fully exploit interactions between modalities, capturing richer patterns. The raw time-series data provides direct, localized signal variations. The GADF image encodes global, nonlinear temporal dependencies. Integrating these multi-granularity information sources offers a more comprehensive basis for final diagnostic decisions.

### 2.3. Data Augmentation Strategies

To address the extreme class imbalance challenge, inspired by strategies for handling imbalanced time-frequency images [23], this study proposes a multi-level data augmentation strategy that specifically targets both time-series data and GADF image data. This approach enhances training set diversity by applying transformations to existing data or generating new synthetic samples.

Unlike image or text data, time-series data possesses an inherent temporal structure that must be preserved during augmentation. Therefore, this study applies the following data augmentation techniques [24] specifically designed for time-series data: jittering, time stretching, amplitude scaling, and slight shifting. The principles of these augmentation methods are described below:

1. Jittering: This technique adds a small, zero-mean Gaussian random value to each data point in the original time series, simulating random noise and minor perturbations commonly found in real-world sensor readings. The jittering formula is as follows:
  (4) $$\tilde{x}(t)=x(t)+\epsilon (t),\epsilon (t)∼N(0,\;\sigma^{2})$$
  where σ controls the noise intensity (typically set to 1–5% of the signal standard deviation).
2. Time Stretching: This technique randomly stretches or compresses segments of a time series to simulate nonlinear temporal deformations in bearing vibration patterns under varying train speeds. It enhances the model’s invariance to changes in time scales.
3. Magnitude Scaling: This technique multiplies the entire time series by a random, small-range scaling factor to simulate global fluctuations in vibration readings caused by external environmental changes or sensor gain variations. It enhances the model’s robustness to variations in signal intensity. The magnitude scaling formula is as follows:
  (5) $$\tilde{x}(t)=\beta \cdot x(t),\beta ∼U(0.9,1.1)$$
4. Time Shifting: This technique randomly shifts the entire time series left or right along the temporal axis by a small number of steps, enabling the model to recognize patterns regardless of their initial position in the sequence.

Within the multimodal framework of this study, image-specific augmentation techniques are applied to GADF images to complement the time-domain strategies applied to raw sensor data. These methods enhance the visual diversity of GADF representations while preserving their structural semantics.

1. Gamma Correction [25]: This technique applies a power-law transformation to adjust the brightness and contrast of GADF images, enhancing the model’s robustness to variations in sensor readings under different lighting or environmental conditions.
2. Histogram Equalization [26]: This technique enhances image contrast by redistributing pixel intensity values, thereby highlighting subtle fault patterns in GADF images that might otherwise be obscured by noise or low contrast.

This dual-strategy data augmentation approach combining time-domain and image-domain techniques enables the generation of more diverse and realistic training data from limited anomalous samples. Furthermore, to address the class imbalance problem, this study adopts an oversampling strategy [27] that utilizes the aforementioned augmentation methods to expand the minority classes, thereby constructing a relatively balanced training set. This significantly enhances the model’s generalization capability.

### 2.4. Self-Adversarial Training

A critical architectural choice in this study is the application of adversarial perturbation to the multimodally fused feature vector, rather than to the raw 1D time-series data or 2D images. This design offers distinct advantages over modality-specific perturbation strategies. If perturbations were applied only to a single modality, the model would learn to resist noise specific to that modality alone. In contrast, our approach directly operates on the fused latent feature space, compelling the model to develop a universally robust feature representation resistant to subtle and complex disturbances from any modality. This strategy not only enables the model to withstand real-world noise that may affect sensor signals or image conversion processes but also forces it to learn more compact and stable features at a deeper level. Consequently, it effectively prevents overfitting and significantly enhances the model’s generalization capability when dealing with unseen, complex real-world data.

**Figure 4:**
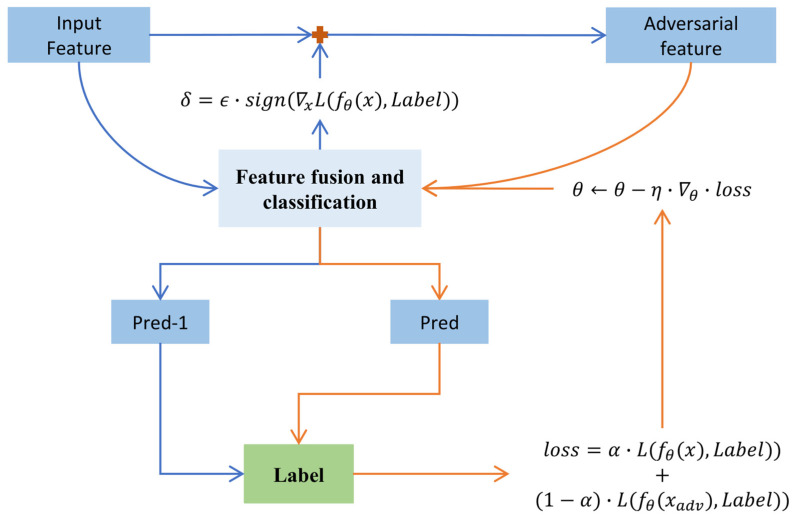
Key Steps of Self-Adversarial Training (SAT). The red plus symbol (“+”) denotes the adversarial perturbation addition operation, where the gradient-based perturbation δ is element-wise added to the original input feature x to generate the adversarial feature $x_{adv}=x+\delta$.

SAT is implemented through an iterative two-phase process [28]: an adversarial attack phase and a conventional training phase. Figure 4 presents a comparative visualization of the data augmentation results.

The adversarial attack phase is executed first. Unlike conventional training, this phase does not update the model weights. Instead, it utilizes gradient information to compute a minimal perturbation, which is then added to the original input data. This perturbation is designed to maximize the model’s loss, thereby generating an adversarial sample capable of “deceiving” the model’s classification.

1. Input and Loss Calculation: Input a clean training sample *x* and its true label *y*. The model performs forward propagation to compute the loss on the clean sample $L(f_{\theta }(x),y)$.
2. Compute Input Gradient: Through backpropagation, calculate the gradient of the loss function with respect to the input $\nabla_{x}L$. This gradient indicates how to subtly adjust *x* to most effectively increase the model’s loss, i.e., make the model more prone to errors.
3. Generate Perturbation: Based on the gradient information, generate an adversarial perturbation:
  (6) $$\delta =\epsilon \cdot sign(\nabla_{x}L(f_{\theta }(x),\;y))$$
  Here, ϵ is a hyperparameter controlling the perturbation intensity (attack magnitude), and sign(·) is the sign function, ensuring the perturbation direction is correct and efficient.
4. Generate Adversarial [25,29] Example: Apply the perturbation to the original sample to obtain the adversarial example $x_{adv}=x+\delta$. This step outputs a “poisoned” adversarial sample $x_{adv}$, which appears almost identical to *x* but causes the current model to misclassify it with high confidence.

This is followed by the conventional training phase, where forward-backward propagation is performed again on the generated adversarial samples. This time, the model weights are updated based on the loss. This forces the model to learn how to correctly classify these “challenging” samples, thereby enhancing its robustness when encountering unseen complex data.

1. Input Adversarial [30] Example: Input the adversarial sample $x_{adv}$ generated in the first phase and its true label *y* into the model.
2. Compute Total Loss: The model simultaneously calculates the loss on both the adversarial sample $L(f_{\theta }(x\_adv),y)$ and the original clean sample $L(f_{\theta }(x),y)$, and computes a weighted sum to obtain the total loss:
  (7) $$L_{total}=\alpha \cdot L(f_{\theta }(x),y)+(1-\alpha )\cdot L(f_{\theta }(x\_adv),y)$$
3. This ensures that the model enhances its robustness while retaining accuracy on the original data.

Backpropagation and Parameter Update: Calculate the gradient of the total loss $L_{totle}$ with respect to the model parameters *θ*, and use an optimizer to update *θ* based on this gradient. The result is a new model with improved “immunity” to the current attack method.

By integrating SAT into the multi-modal training process, we expect the model to not only benefit from data augmentation but also further enhance its generalization performance in real non-stationary, high-noise environments through learning to resist adversarial perturbations.

## 3. Case Study

In this section, the proposed multimodal fusion and SAT framework is evaluated using operational data from train bearings. Through comparison with typical baseline models and ablation experiments, the effectiveness of the method in handling class imbalance and complex operating conditions is validated.

### 3.1. Datasets and Experimental Setup

The experimental dataset utilized in this study is derived from real-world operational data collected from a railway vehicle, rather than a laboratory test rig. This ensures the data reflects the complex, non-stationary characteristics of actual railway environments. The vibration signals were acquired using high-precision vibration sensors (accelerometers) mounted on the axle boxes of the train bogies. The data acquisition system monitored the vibration acceleration (measured in m/s^2^) across axles 1 through 8 to ensure a comprehensive representation of the train’s running state.

The sampling frequency was set to 12.8 kHz, which is sufficient to capture the characteristic fault frequencies of the bearings. The data collection spanned a wide range of operational speeds, varying from 0 to 120 km/h. This speed variation introduces significant non-stationarity to the signals, covering different phases including acceleration, constant speed cruising, and deceleration. Consequently, the dataset inherently includes noise and interferences caused by wheel–rail interactions, track irregularities, and varying load conditions, making the anomaly detection task significantly more challenging and realistic compared to standard open datasets.

#### 3.1.1. Data Preprocessing and Statistical Reliability Verification

The raw vibration signals were acquired at a sampling frequency of 12.8 kHz. Due to the complex rail-wheel interactions, particularly the impact when passing over rail gaps, the raw time-series data frequently exhibits high-amplitude impulsive spikes. These occasional spikes act as outliers that can distort the feature distribution and negatively affect model training. To address this, we employed an improved Hampel filtering strategy combined with linear interpolation. Unlike standard low-pass filters that might blur valid high-frequency fault features, this method uses a sliding window to calculate the local median and standard deviation (σ). Data points exceeding the μ±3σ threshold are identified as outliers caused by rail joint disturbances. These outliers are then replaced using linear interpolation based on neighboring points, rather than simple median replacement, to preserve the continuity and trend of the signal.

To further ensure the quality and statistical reliability of the processed data, and to verify that the dataset is free from significant stochastic anomalies that could bias the experimental results, we applied the Grubbs’ test and the Romanovsky criterion to evaluate data homogeneity.

1. Grubbs’ Test for Outliers: This criterion was used to detect any remaining single outliers in the segmented samples. The calculated G-statistic for the normal operating data was found to be consistently below the critical value at a significance level of α=0.05, indicating that no significant outliers remained after preprocessing.
2. Romanovsky Criterion for Homogeneity: Given the limited number of anomaly samples, the Romanovsky criterion (*t*-test based) was utilized to assess the homogeneity of the variance across different data segments. The results demonstrated that the calculated test statistics for the vast majority of samples fell within the acceptance region $R_{y}=[1,\;3]$, confirming that the data possesses stable statistical properties and is suitable for training deep learning models.

These rigorous preprocessing and statistical validation steps confirm that the experimental dataset is reliable, homogeneous, and that the impact of random environmental factors has been effectively minimized.

**Figure 5:**
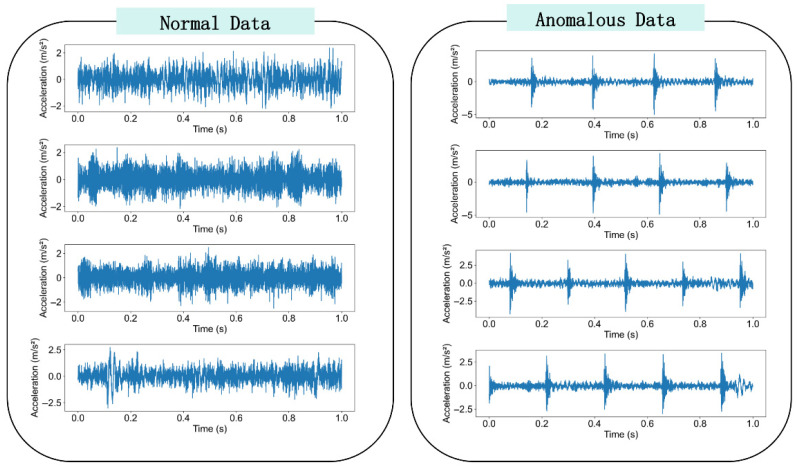
Samples of normal data and anomalous data.

After data filtering and segmentation, a total of 16,241 normal data samples and 227 anomalous data samples were obtained. Due to the rarity of bearing anomalies, the dataset exhibits severe class imbalance, a core challenge this framework aims to address. The time series in the dataset vary in length, and the bearing vibration data are influenced by multiple external conditions (e.g., train speed, ambient temperature, and travel distance), making the diagnostic task highly challenging. Samples of the processed data are shown in Figure 5.

#### 3.1.2. Data Partitioning and Class Balancing Strategy

To ensure a rigorous evaluation of the model’s generalization capability, the preprocessed dataset was randomly divided into training, validation, and testing sets following a 60%:20%:20% ratio. As detailed in Table 1, the original dataset exhibits a severe class imbalance, with a ratio of normal to anomalous samples of approximately 71:1. Such disparity often leads to models that are biased towards the majority class.

To address this, the proposed composite data augmentation strategy (including jittering, time stretching, and GADF enhancement) was applied. It is crucial to note that data augmentation was strictly limited to the training set to prevent data leakage and ensure that the validation and testing sets consist solely of real-world operational data. As shown in the “Augmented” column of Table 1, we generated synthetic anomaly samples to balance the training set, achieving a near 1:1 ratio between normal and anomalous classes during the training phase.

#### 3.1.3. Hyperparameter Optimization

The performance of deep learning models is highly sensitive to hyperparameter settings. To obtain the optimal configuration for the proposed Multimodal SAT framework, we conducted a systematic grid search on the validation set. The optimization focused on key parameters including the learning rate, batch size, and the adversarial perturbation coefficient (*ϵ*) specific to the SAT mechanism.

The search ranges and the final optimized values are presented in Table 2. The model was trained using the Adam optimizer, with an early stopping mechanism triggered if the validation loss did not decrease for 10 consecutive epochs.

### 3.2. Comparison Method and Evaluation Metrics

To thoroughly evaluate the performance of the proposed framework, we compare it with traditional machine learning approaches and single-modal deep learning models. The baseline models are established as follows:

1. Baseline 1 (SVM): A Support Vector Machine model based on manual feature extraction, representing the traditional classification paradigm.
2. Baseline 2 (1D CNN): A 1D CNN model trained directly on raw time-series data.
3. Baseline 3 (2D CNN): A 2D CNN model trained on GADF image data.

The following standard metrics are adopted to assess model performance: Accuracy, Precision, Recall, and F1-score. The F1-score is particularly important for imbalanced datasets, as it balances the trade-off between precision and recall.

### 3.3. Analysis of Experimental Results

This section presents a qualitative validation of the data processing modules followed by a quantitative analysis of the model’s classification performance.

#### 3.3.1. Visualization of Data Processing and Augmentation

To qualitatively validate the effectiveness of the proposed preprocessing and data augmentation modules, we visualized the intermediate data states.

**Figure 6:**
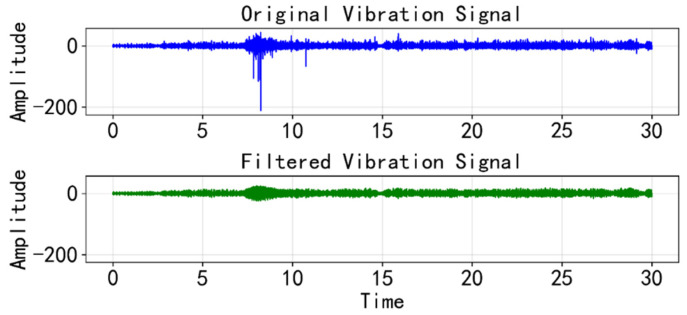
Samples of data before and after filtering.

First, Figure 6 presents the comparison between the raw vibration signal and the signal processed by the improved Hampel filter. As shown in the zoomed-in view, the raw signal contains sharp, non-periodic impulsive noise caused by rail gap impacts. The filtering process effectively suppresses these outliers while preserving the original trend and fault characteristic frequencies, ensuring cleaner input for the model.

**Figure 7:**
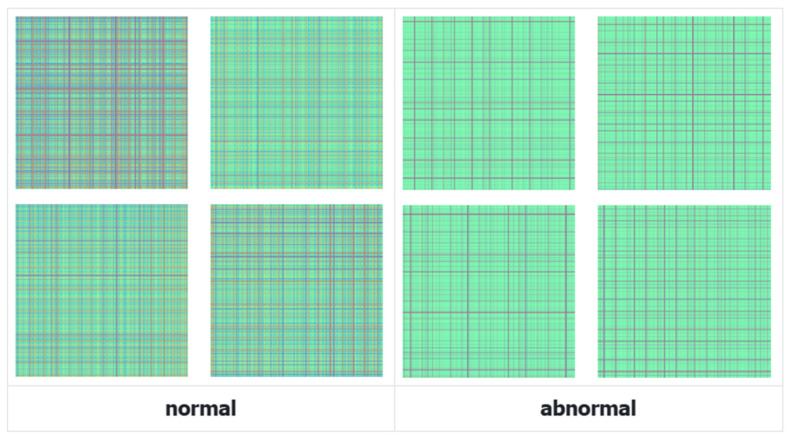
Samples of normal and abnormal GADF diagrams.

Figure 7 illustrates the generated GADF images. Compared to the raw 1D time series, the GADF images effectively encode the temporal correlations into distinct texture patterns. The clear structural differences between normal and anomalous states in the GADF domain provide the 2D CNN branch with rich discriminative features.

**Figure 8:**
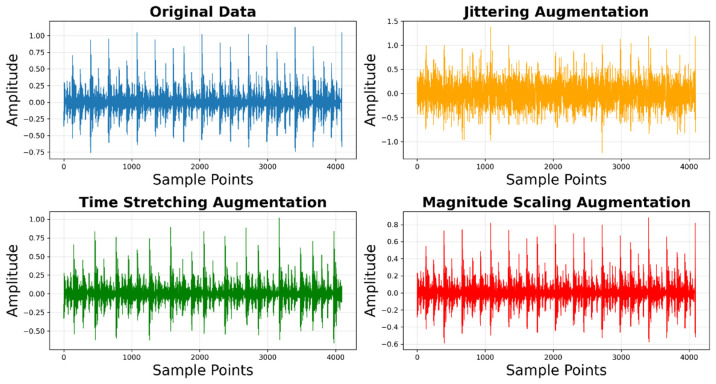
Samples of time series data augmentation.

**Figure 9:**
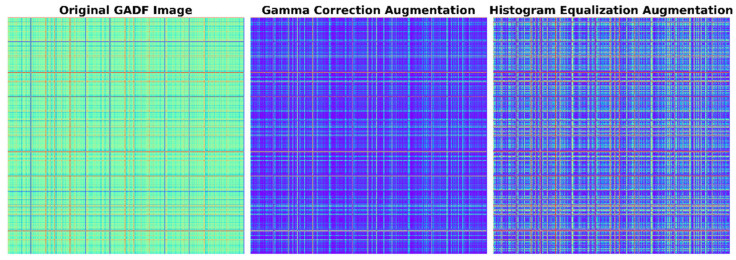
Samples of image data augmentation.

Finally, Figure 8 and Figure 9 demonstrates the diversity introduced by the data augmentation strategy. By applying time stretching and jittering, the original minority class samples are transformed into physically consistent variations. This visual confirmation ensures that the augmented samples are not mere duplicates but valid expansions of the feature space.

#### 3.3.2. Comparative Performance and Classification Details

To evaluate the model’s performance reliability, each model was trained and tested independently 8 times using different random seeds.

As shown in Table 3, the proposed method outperforms all baselines with the highest accuracy (99.73%) and the lowest standard deviation (±0.05%), demonstrating superior stability.

**Figure 10:**
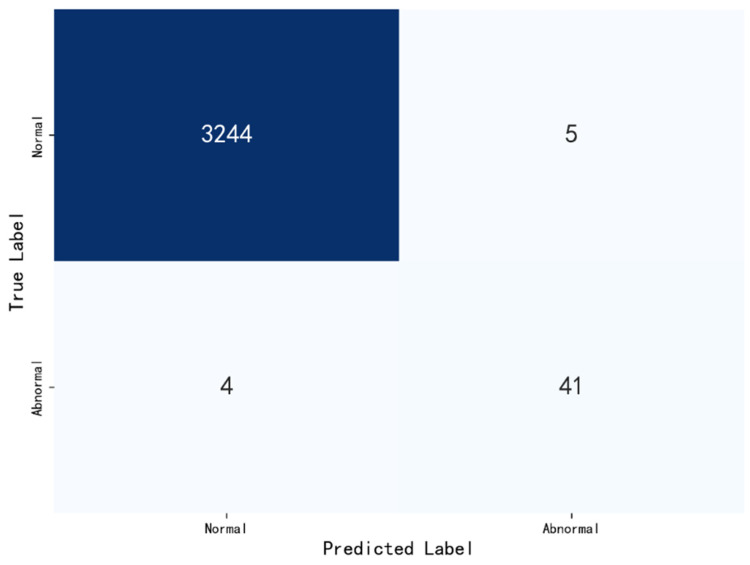
Samples of confusion matrix diagram.

To further analyze the classification details, particularly regarding the class imbalance issue, we visualized the prediction results using a Confusion Matrix (as shown in Figure 10). In railway anomaly detection, “False Negatives” (missing a true fault) are considered the most critical error. The confusion matrix shows that our model achieves a high correct classification rate for the minority “Anomaly” class, with very few fault samples misclassified as normal.

### 3.4. Ablation Validation and Robustness Analysis

#### 3.4.1. Ablation Validation

To further verify the effectiveness of specific modules, specifically data augmentation and SAT, ablation experiments were conducted. The results are shown in Table 4.

Effectiveness of Data Augmentation: As observed in Table 4, the introduction of augmentation techniques (jittering, time stretching, amplitude scaling) significantly improves generalization compared to the baseline trained solely on raw data. This confirms that the designed strategy effectively simulates non-stationary interference and increases data diversity, preventing overfitting to limited anomalous samples.

Analysis of the SAT Mechanism: The addition of SAT further enhances performance (F1-score increases to 94.98%). Unlike traditional adversarial training which may harm minority classes in imbalanced datasets, the proposed framework leverages multimodal fusion to create a robust and “separable” feature space. In this optimized space, SAT forces the model to learn more generalized features by resisting perturbations, rather than exacerbating inter-class disparities.

#### 3.4.2. Noise Robustness Analysis

In real-world railway environments, acquired signals are inevitably corrupted by various background noises (e.g., wheel–rail friction, aerodynamic noise). Although the proposed method performs well on the original test set, it is crucial to evaluate its robustness under varying noise intensities.

To verify this, we introduced additive Gaussian white noise to the standardized test set samples to construct composite signals with different Signal-to-Noise Ratios (SNR). The SNR is defined as:

(8) $$SNR_{dB}=10{log}_{10}\left(\frac{P_{signal}}{P_{noise}}\right)$$

where $P_{signal}$ and $P_{noise}$ represent the power of the signal and noise, respectively. We evaluated the F1-score of the proposed method and the three baseline models across a wide SNR range from −4 dB to 10 dB.

**Figure 11:**
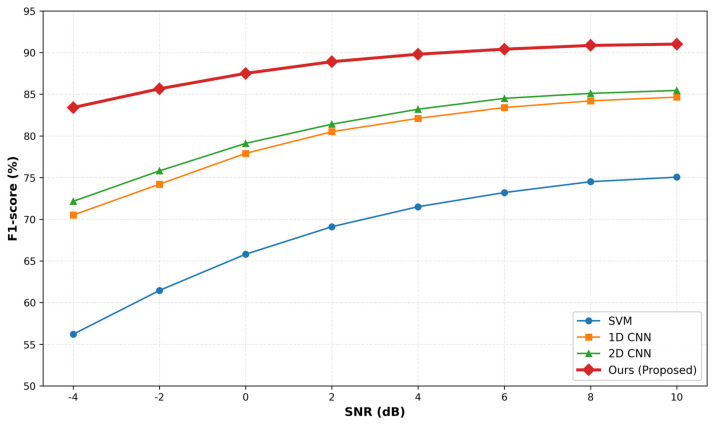
Model prediction results at different noise levels.

Figure 11 illustrates the performance degradation trends of different models as the noise level increases (i.e., SNR decreases).

As observed in Figure 11, the performance of all models declines as the environment becomes noisier. However, the degradation rates differ significantly:

1. Baseline 1 (SVM) exhibits the poorest robustness, with its F1-score dropping sharply below 60% when SNR < 0 dB, indicating its inability to handle high-noise scenarios.
2. Baseline 2 and 3 (Unimodal CNNs) show moderate robustness but still experience a noticeable performance drop (over 12% loss) in high-noise conditions (−4 dB).
3. Proposed Method demonstrates superior stability. Even under the harshest condition (SNR = −4 dB), it maintains an F1-score above 83%.

This exceptional robustness is attributed to the SAT mechanism. By exposing the model to “worst-case” perturbations during the training phase, SAT effectively forces the model to ignore non-robust features (like random noise) and focus on the intrinsic, invariant patterns of the bearing faults, ensuring reliable operation in noisy industrial environments.

## 4. Conclusions and Future Work

### 4.1. Conclusions

This study proposes a strategy based on multimodal fusion and SAT for anomaly detection in train bearing vibration time-series data. The method effectively addresses the challenges of limited samples and signal complexity by integrating raw time-series signals with their GADF images at the feature level, supplemented by a dual time-domain and image-domain data augmentation strategy. Furthermore, the introduced SAT mechanism significantly enhances the model’s robustness, enabling it to maintain high performance even in noisy and unfamiliar environments. Quantitative experimental evaluations demonstrate the superiority of the proposed framework. Specifically, the model achieves an F1-score of 90.11%. Compared to the best-performing unimodal baseline (2D CNN), the proposed method improves the F1-score by 4.66%. Moreover, robustness analysis confirms that the model maintains an F1-score exceeding 83% even under harsh noise conditions (SNR = −4 dB), highlighting the approach’s strong potential for future applications in intelligent railway maintenance.

### 4.2. Limitations

Despite the promising results, certain limitations of the proposed study should be acknowledged. First, the computational cost is relatively high. The process of converting time-series data into GADF images and training a dual-branch multimodal network requires significantly more memory and computational resources compared to lightweight 1D-CNNs. Additionally, the iterative nature of the SAT mechanism extends the model training time. Second, while the data augmentation strategy effectively handles class imbalance, the model’s generalization capability to completely unseen fault types (those with physical characteristics vastly different from the training anomalies) remains to be verified. Finally, although visualization techniques have been employed, the model remains fundamentally data-driven. The lack of direct mapping between the learned deep features and specific physical failure mechanisms (such as bearing kinematic frequencies) may still pose challenges for acceptance in safety-critical engineering applications without further physics-guided validation.

### 4.3. Future Work

While this study has achieved encouraging results, several directions warrant further exploration:

1. Multi-sensor Fusion: Future work could explore integrating bearing vibration data with other sensor data (e.g., vibration, acoustic emissions, or motor current) to develop a more comprehensive diagnostic system. This would provide richer anomaly information, enabling earlier and more accurate anomaly identification.
2. Real-time Deployment: Research is needed to lightweight the proposed framework for real-time inference on onboard or edge devices.
3. Transfer Learning: Investigate transfer learning or domain adaptation techniques to enable the model to rapidly transfer knowledge learned from one train type or operational condition to unseen scenarios, reducing deployment costs across different railways or locomotives.
4. Interpretability: Despite the superior performance of data-driven models, their “black-box” nature remains a concern in safety-critical fields. Future research should focus on enhancing the interpretability of model decisions, such as validating features learned by deep learning models through physical models, thereby increasing their credibility in industrial applications.

## Figures and Tables

**Table 1:** Distribution of samples across datasets before and after augmentation.

Dataset Subset	Class	Original Samples	Augmentation Factor	Final Samples	Description
Training Set (60%)	Normal	9745	None	9745	Real Data
	Anomaly	137	×71 (approx.)	9745	Real + Synthetic
Validation Set (20%)	Normal	3248	None	3248	Real Data
	Anomaly	45	None	45	Real Data
Test Set (20%)	Normal	3248	None	3248	Real Data
	Anomaly	45	None	45	Real Data

**Table 2:** Hyperparameter optimization ranges and final selected values.

Hyperparameter	Description	Search Range	Optimized Value
Batch Size	Number of samples per gradient update	{32,64,128}	64
Learning Rate	Initial learning rate for Adam optimizer	$\left\{1\times 10^{-4},5\times 10^{-4},1\times 10^{-3},5\times 10^{-3}\right\}$	$1\times 10^{-3}$
Epochs	Maximum training iterations	{50,100,150}	100
SAT Perturbation (ϵ)	Intensity of adversarial noise	{0.001,0.01,0.05,0.1}	0.01
Loss Weight (α)	Weight trade-off in SAT total loss	{0.3,0.5,0.7}	0.5
Dropout Rate	Probability of neuron deactivation	{0.2,0.3,0.5}	0.5

**Table 3:** Performance comparison with baseline models.

Model	Accuracy (%)	Precision (%)	Recall (%)	F1-Score (%)
Baseline 1 (SVM)	76.57±1.24	75.20±1.35	74.90±1.18	75.05±1.22
Baseline 2 (1D CNN)	86.20±0.85	86.80±0.92	87.50±0.81	84.65±0.88
Baseline 3 (2D CNN)	87.95±0.76	86.60±0.68	97.30±0.72	85.45±0.75
Ours (Proposed)	99.73±0.05	89.13±0.38	91.11±0.45	90.11±0.41

**Table 4:** Experiments on data augmentation and SAT ablation.

Configuration	Accuracy (%)	Precision (%)	Recall (%)	F1-Score (%)
Baseline	99.73	89.13	91.11	90.11
Data Augmentation	99.78 (+0.05)	91.54 (+2.41)	94.4 (+3.29)	92.96 (+2.85)
Data Augmentation+ SAT	99.85 (+0.07)	93.04 (+3.91)	96.81 (+5.70)	94.98 (+4.87)

## Data Availability

The datasets presented in this article are not readily available because they contain privacy-sensitive information that cannot be shared. Requests to access the datasets should be directed to the corresponding author.
